# Asymmetric dispersal structures a riverine metapopulation of the freshwater pearl mussel *Margaritifera laevis*

**DOI:** 10.1002/ece3.1135

**Published:** 2014-07-05

**Authors:** Akira Terui, Yusuke Miyazaki, Akira Yoshioka, Kenzo Kaifu, Shin-ichiro S Matsuzaki, Izumi Washitani

**Affiliations:** 1 Department of Ecosystem Studies, Graduate School of Agricultural and Life Sciences, University of Tokyo1-1-1 Yayoi, Bunkyo-ku, Tokyo, 113-8657, Japan; 2 Department of Forest Science, Graduate School of Agriculture, Hokkaido UniversityKita 9, Nishi 9, Kita-ku, Sapporo, 060-8589, Japan; 3 Kanagawa Prefectural Museum of Natural History499 Iryuda, Odawara, Kanagawa, 250-0031, Japan; 4 National Institute for Environmental StudiesTsukuba-shi, Ibaraki, 305-8506, Japan; 5 Faculty of Law, Chuo UniversityHachioji-shi, Higashi-nakano 742-1, Tokyo, 192-0393, Japan

**Keywords:** Dendritic networks, mutualism, running water, Unionoida

## Abstract

Unidirectional water flow results in the downstream-biased, asymmetric dispersal of many riverine organisms. However, little is known of how asymmetric dispersal influences riverine population structure and dynamics, limiting our ability to properly manage riverine organisms. A metapopulation of the freshwater pearl mussel *Margaritifera laevis* may be sensitive to river currents because mussels are repeatedly exposed to downstream drift during floods—a parasitic life stage is the only, limited period (∼40 days) during which larvae (glochidia) can move upstream with the aid of host fish. We hypothesized that water-mediated dispersal would overwhelm upstream dispersal via host fish, and therefore, that upstream subpopulations play a critical role as immigrant sources. To test this hypothesis, we examined the effects of both up- and downstream immigrant sources on the size of target subpopulations in the Shubuto River system, Hokkaido, Japan. We found that target subpopulation size was dependent on the upstream distribution range of reproductive subpopulations and the number of upstream tributaries, which are proxies for the number of potential immigrants moving downstream. In contrast, little influence was observed of downstream immigrant sources (proximity to downstream reproductive subpopulations). These results were consistent even after accounting for local environments and stream size. Our finding suggests that upstream subpopulations can be disproportionately important as immigrant sources when dispersal is strongly asymmetric.

## Introduction

Metapopulation theory has invoked the importance of spatial dynamics in long-term population persistence (Hanski 1999), and this view is increasingly accepted for lotic organisms such as salmonids (Dunham and Rieman 1999; Koizumi and Maekawa 2004; Isaak et al. 2007). An inherent property of most metapopulations is variation in subpopulation size, such that individual subpopulations may contribute unequally to metapopulation persistence (e.g., Foppen et al. 2000; MacPherson and Bright 2011). In patchy habitat systems, this contribution has often been predicted by habitat size and isolation, implicitly assuming many possible dispersal pathways and symmetric dispersal among subpopulations (e.g., Hanski 1994; Kuroe et al. 2011). These assumptions, however, may be violated in many lotic organisms, for which suitable habitats represent hierarchical branching geometries (dendritic ecological networks, or DENs; sensu Grant et al. 2007) and dispersal processes are influenced by the unidirectional nature of water flow (Alp et al. 2012; Altermatt 2013).

A growing body of theoretical evidence suggests that directionally biased dispersal (asymmetric dispersal) has negative impacts on metapopulation persistence because it produces only “donor” or “recipient” subpopulations within a metapopulation (Vuilleumier and Possingham 2006). This prediction implies an inherent vulnerability of riverine metapopulations and is supported by recent theoretical studies focusing on metapopulation dynamics in DENs (Grant 2011; Mari et al. 2014; Yeakel et al. 2014). However, our ability to predict and manage riverine metapopulations is limited by the lack of empirical knowledge about how unidirectional water flow, together with the structural constraints of dendritic networks, delineates the spatial features of metapopulations. This understanding would help to identify those subpopulations most critical for the persistence of riverine metapopulations and is particularly relevant to high-gradient streams where the dispersal of less-mobile organisms may be extremely biased downstream.

The endangered freshwater pearl mussel *Margaritifera laevis*, classified as vulnerable in Japan (Government of Japan 2013), is a long-lived (maximum life span 79 years; Awakura 1969) species inhabiting high-gradient Japanese streams. As with other unionoids, mussels that stably settle to the bottom (hereafter “settled mussels”) often occur as discrete, dense aggregations called mussel beds, which represent subpopulations (hereafter “settled subpopulation”) within a riverine metapopulation (Strayer 2008). The dynamics of this metapopulation may be under the influence of strongly asymmetric dispersal because mussels are repeatedly exposed to downstream drift by floods (Hastie et al. 2001; Kurihara and Goto 2011)—an early parasitic life stage is the only, limited period (∼40 days) during which larvae (glochidia) can move upstream (and/or downstream) with the aid of the obligate host fish *Oncorhynchus masou masou* (masu salmon; Terui et al. 2014). Consequently, the state of mussels in a postparasitic life stage (i.e., after leaving host fish as juvenile mussels) can be organized into settled and unsettled phases.

In the settled phase, settled subpopulations are formed only in suitable local environments (Strayer 2008). Stable areas of the river even under high flows may enable postparasitic mussels to stably settle to the substrate (Morales et al. 2006; Allen and Vaughn 2010), but their survival rate following the settlement is probably influenced by local habitat quality, including simple hydraulic conditions (e.g., water depth), bed materials, and water quality (Geist and Auerswald 2007; Strayer 2008; Terui et al. 2011; Strayer and Malcom 2012). These processes are likely to cause the patchy distribution of settled subpopulations, allowing us to directly evaluate their demographic and spatial status. Alternatively, postparasitic mussels in the unsettled phase (hereafter, “unsettled mussels”) may be distributed diffusely throughout the range of host fish and may ultimately immigrate to settled subpopulations downstream. Given the tiny body size of metamorphosed juvenile mussels (<0.7 mm in shell length; Kondo 2008) and their low growth rate (<10 mm per year; Akiyama and Iwakuma 2009), the species may drift for some time following the limited period of host-mediated dispersal. Under these circumstances, water-mediated dispersal of unsettled mussels would overwhelm an influence of immigration from downstream via host-mediated dispersal, and thus, upstream subpopulations may play a critical role as immigrant sources.

To test this hypothesis, we examined the effects of both up- and downstream immigrant sources on the size of settled subpopulations. To quantify the upstream immigrant sources, one must indirectly estimate the distribution range of potential fish-dispersed immigrants, as unsettled mussels may include “invisible” tiny juveniles immediately after dropping from host fish. Two proxies can be useful in this situation: the upstream distribution range of potential immigrants (UDR) and the number of upstream confluences (NUC). The UDR should reflect the number of postparasitic immigrants moving downriver, after originating from upstream settled subpopulations. The NUC should correlate positively with immigrant source size since *O. masou masou* infected with glochidia prefer cooler tributaries as thermal refugia during summer (Terui et al. 2014). To assess downstream immigrant sources, we examined proximity to downstream reproductive subpopulations. At the same time, we also considered an influence of local environments, including substrate instability and local habitat quality. Substrate instability under high flows may influence the size of settled subpopulations through affecting immigration success of unsettled mussels and/or emigration of settled mussels, while local habitat quality (simple hydraulics, bed materials, and water quality) seems to be related to survival rate of settled mussels (Terui et al. 2011). We focused on *M. laevis* populations in the Shubuto River system, Hokkaido, Japan, where the relatively undisturbed environment allowed us to test the importance of the metapopulation processes for *M. laevis* (Miyazaki et al. 2011; Terui et al. 2011).

## Methods and Materials

### Study area and study species

Investigations were conducted in the Shubuto River system near Kuromatsunai, Hokkaido Prefecture, Japan (42°40′N, 140°18′E), where the mean annual temperature and mean annual precipitation are 7.4°C and 1682.8 mm, respectively (averaged for 2010–2013; Japan Meteorological Agency 2014). The water catchment area encompasses 367 km^2^ of forested and mountainous terrain, and the length of the mainstem is ∼40 km. No dams or weirs prevent migration of host fish in the mainstem, although some small weirs (height < 5 m) are present in the upstream reaches of tributaries. The host fish was abundant and widely distributed throughout the river system (Miyazaki et al. 2011) and spatial variation in host fish density is probably too small to have a direct impact on host- and water-mediated immigration processes. Water quality was suitable for most freshwater organisms (dissolved oxygen >95% in degrees of saturation, biochemical oxygen demand 0.5–1.7 mg/L, and ammonia concentration <0.05 mg/L; Terui et al. 2011; Kuromatsunai Town 2014).

In the Shubuto River system, glochidia of *M. laevis* are released in the early summer, from early to mid-July, and infect the gills of host fish (mainly parrs) with an extremely high prevalence near dense mussel beds (∼100%; Terui et al. 2014). This parasitic stage lasts for approximately 40–50 days (Kondo 2008). Juveniles with shell lengths ranging from 0.3 to 0.6 mm detach from the host fish during late summer (Kondo 2008; Terui et al. 2014) and disperse passively via river currents. As released glochidia can survive for only a few days (<2 days) with summer water temperature (>15°C) before they must find a suitable host fish (otherwise they die; Akiyama and Iwakuma 2007), water-mediated dispersal of *M. laevis* is essentially limited to the postparasitic life stage. Sexual maturity occurs at 8–13 years of age (Akiyama 2007; Kondo 2008), and maximum life span is ∼79 years (Awakura 1969; Kondo 2008). *Margaritifera laevis* is the only species of freshwater mussel within the riverine network, and the species has no known predators (e.g., crayfish, muskrats).

We first conducted spatially continuous surveys for reproductive subpopulations throughout the river system during the summer and fall of 2010–2013. Subsequently, we conducted quantitative surveys of mussel subpopulations, substrate stability, and local habitat variables during the spring and summer of 2010–2013.

### Distribution ranges of reproductive subpopulations

**Figure 1:**
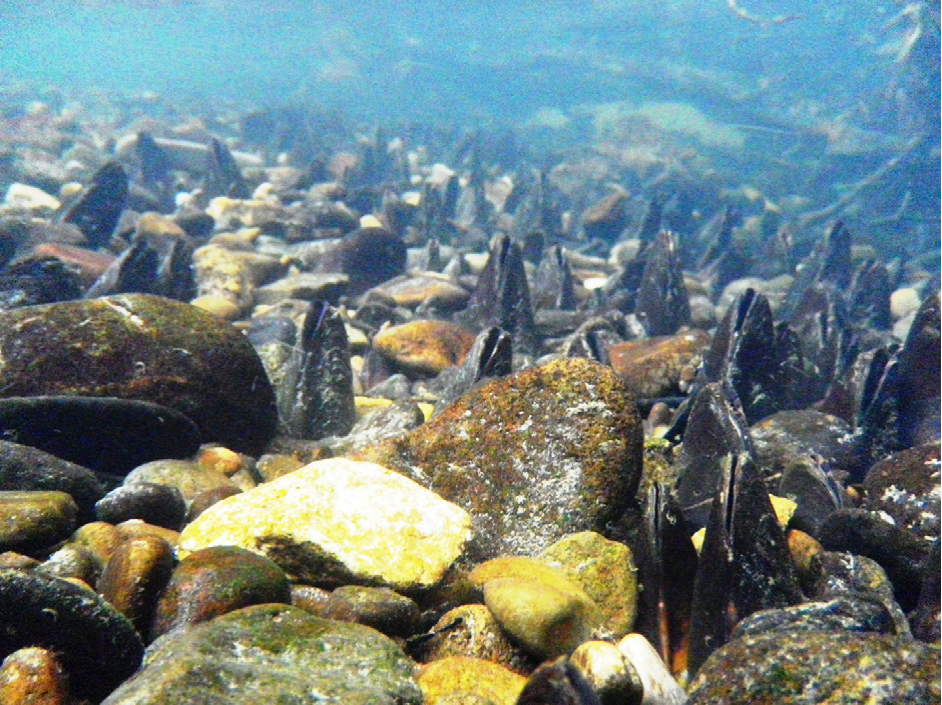
Discrete, dense aggregation of *Margaritifera laevis* called mussel beds, which represent subpopulations within a riverine metapopulation.

Spatially continuous surveys were conducted to assess the distribution range of reproductive subpopulations throughout the Shubuto River system. This information was used to summarize the *M. laevis* metapopulation and to estimate the UDR, NUT, and proximity to downstream reproductive subpopulations (see Proxy variables for immigrant sources). In this study, subpopulations were defined as aggregations of mussels located within 20 m of each other. This distance reflects the maximum spatial extent of a mussel bed (A. Terui, pers. obs.), as well as local recruitment via host fish (Terui et al. 2014). Our preliminary survey showed that no fish were infected with significant numbers of glochidia (>10 glochidia/fish—glochidial survival rate on fish is <10%; Bauer and Wächtler 2001) near any mussel bed with a density of ≤15 adult mussels (ind.)/m^2^ (Terui 2014). Based on this information, we defined a mussel bed with a density of >15 ind./m^2^ as a reproductive subpopulation. Reproductive subpopulations are easy to detect while wading and snorkeling because they form large, visible aggregations (Fig. 1).

**Figure 2:**
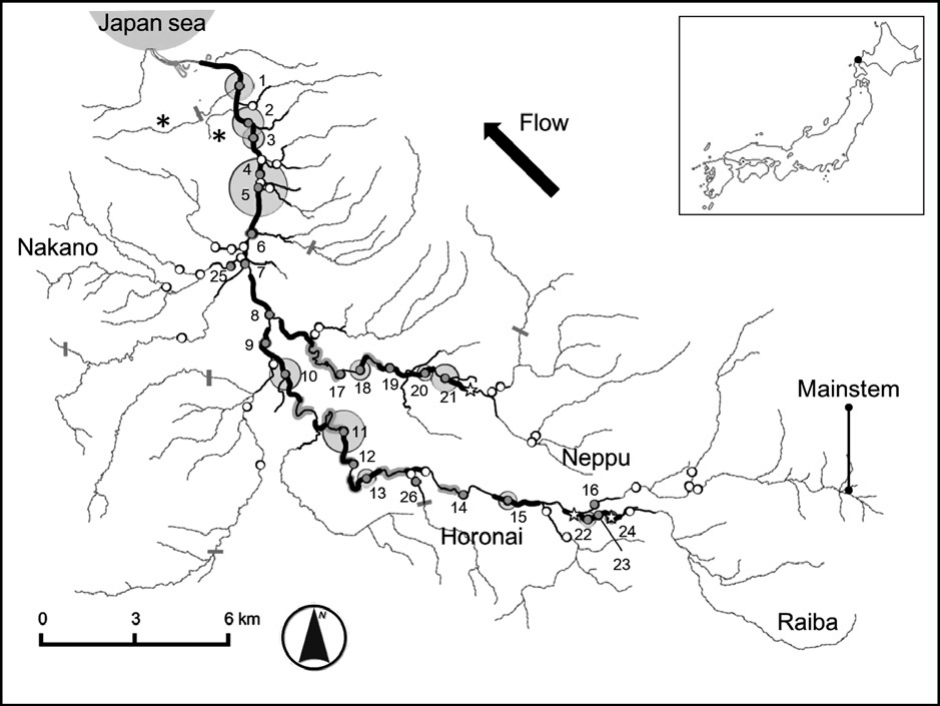
Map of the Shubuto River system. Filled and open plots indicate sampling sites with and without mussels, respectively. The size of bubbles is proportional to the settled subpopulation size (range: 1–792 mussels per site). Numbers by filled plots indicate the subpopulation ID (see also Fig. 3). Reaches (0.5 km) with reproductive subpopulations are shown in thick black lines. Thin black lines represent the distribution range of potential immigrants estimated from host fish dispersal. Stars indicate the upstream range limits of reproductive subpopulations in each river. Gray-shaded river lines indicate river sections that were inaccessible during spatially continuous surveys. Thick gray lines crossing rivers represent river-crossing structures preventing dispersal of *Oncorhynchus masou masou*. Note that the Yunosawa and Mitsutaki streams (asterisks) were excluded from the distribution range of potential immigrants.

We started our surveys at the Shubuto mainstem and Neppu rivers, which are known to support mussel populations (N. Hatai, pers. comm.). Surveys were conducted by at least two investigators and progressed upstream from each river mouth until encountering a dispersal barrier for *O. masou masou*, or a reach with cascade or step-pool structures where no mussel beds were found in previous surveys (Terui et al. 2011). In the same manner, we subsequently waded into small tributaries that flow into the Shubuto mainstem and Neppu rivers. We recorded the presence/absence of reproductive subpopulations at a resolution of 0.5 km. The upstream range limits of reproductive subpopulations in each stream were located using a global positioning system (GPS) device (Colorado 300; Garmin international, Inc., Olathe, KA) during the surveys. Some reaches were not accessible because of swift currents and/or extreme scouring (Fig. 2).

### Quantitative surveys of mussel subpopulations

We selected 57 sampling sites (18 sites in 2010, 28 sites in 2011, and 11 sites in 2012) within the riverine network, whose spatial scale (20 m length) was intended to coincide with the spatial extent of *M. laevis* subpopulations (see above). Sampling sites were distributed so as to encompass the entire riverine network and were separated from each other by at least 300 m in watercourse distance, excluding reaches with concrete riverbeds, cascade or step-pool structures, and/or extreme scouring.

At each site, we recorded site coordinates by GPS and established four equally spaced transects across the river. Within each transect, we placed three 0.25-m^2^ quadrats, one at mid-channel and another at each side, near the bank, for a total of 12 quadrats per site. We collected mussels (e.g., settled mussels) from each quadrat as follows. After all visible mussels had been censused using a glass-bottomed viewing bucket, we excavated the mussel bed to a depth of ∼15 cm using a trowel and immediately sieved all material through a 2-mm mesh sieve. In our preliminary survey, mussel abundance at each site was strongly correlated with subpopulation size (Pearson's *r* = 0.89, *P *< 0.001, *n* = 10), which was obtained by multiplying mean mussel density (averaged for 2–30 quadrats depending on mussel bed size) by mussel bed area. This indicates that mussel abundance per site is a suitable proxy for subpopulation size.

After sampling, digital images of all sampled mussels were captured alongside a ruler using an Optio WS80 camera (Pentax, Tokyo, Japan). These were subsequently analyzed using ImageJ (National Institutes of Health, Bethesda, MD) to calculate mussel shell length in millimeters and then to discern between adult (≥50 mm in shell length; Kondo 2008) and juvenile mussels (<50 mm). We released all sampled mussels back into the sampling quadrat of origin immediately after completing photography.

Shell length distribution at each settled subpopulation was measured to reinforce statistical inference of water- and host-mediated immigration (see also Statistical analysis). For example, one would be expected that smaller (or younger) mussels (soon after dropping from host fish) may occur in a wider range of habitats than larger (or older) mussels if water- and host-mediated dispersals are indeed functioning.

### Proxy variables for immigrant sources

The distribution range of potential immigrants throughout the river network was estimated by combining the known dispersal distance of *O. masou masou* with the distribution of reproductive mussel subpopulations. Direct estimates of *O. masou masou* dispersal are rare, but the longest reported dispersal distance is ∼1500 m during *M. laevis*' parasitic stage (Sakata et al. 2005). Accordingly, we estimated the distribution range of potential immigrants with the assumption that glochidia potentially disperse 1500 m upstream from the nearest reproductive subpopulation (see Fig. 2). This assumption was supported by our observations that no fish infected with glochidia were found >1500 m upstream from the nearest reproductive subpopulation (Terui 2014). Subsequently, the UDR, a proxy for upstream immigrant source size, was estimated as follows:
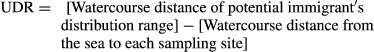
(1)

When the estimated UDR was negative (i.e., the sampling site is located outside the distribution range of potential immigrants), we treated them as zero because it has virtually no potential immigrants from upstream reaches.

In determining the NUC, we considered only confluences of tributaries that connect directly to the rivers in which each sampling site was located. We did not count confluences that were located outside the distribution range of potential immigrants. Because the exact drifting distance of unsettled *M. laevis* is unknown in the Shubuto River system, the NUC was considered at three distance classes: 1, 2, and 3 km upstream (watercourse distance) from each sampling site. Upstream reaches fragmented by dams or weirs were removed from the UDR and NUC calculations. We also excluded the Yunosawa and Mitsutaki streams from our calculations because no *O. masou masou* were found there (asterisks in Fig. 2; Miyazaki et al. 2013).

Proximity to downstream reproductive subpopulations was determined based on the presence/absence of a reach containing reproductive subpopulations within 1500 m downstream from the target location. The distance under consideration was determined by the dispersal capability of *O. masou masou*. We tentatively treated any reaches that were inaccessible due to swift currents and/or extreme scouring as unoccupied because they appeared unsuitable for reproductive subpopulations. This did not qualitatively alter the results. The UDR, NUC, and proximity to downstream reproductive subpopulations were calculated using ArcGIS 10.0 (Esri, Redlands, CA) with Digital Map 25000 (National and Regional Planning Bureau), at a scale of 1:25,000.

### Substrate stability under bankfull flow conditions

Substrate stability under high-flow conditions is a particularly important factor affecting mussel abundance and species richness (e.g., Allen and Vaughn 2010). We evaluated substrate stability based on shear stress (τ_0_), in which higher values represent lower stability (Lorang and Hauer 2003). To calculate shear stress, we gathered data on channel geomorphology, including channel cross-sectional area, wetted perimeter, and water surface slope. Because measuring channel geomorphology directly at bankfull flow levels was impossible (bankfull refers to the water level just before spilling out of the channel), we gathered data indirectly as follows.

First, we visited each sampling site during a period of bankfull flows (3–5 May 2013; average discharge = 48.1 m^3^/s) and placed a metal peg at the water's edge, allowing us to accurately measure the water level later. We also measured bankfull river width with a laser distance meter (TruPulse 200; Laser Technology Inc., Centennial, CO).

Second, we returned to each sampling site during a period of low flows (19–25 June 2013; average discharge = 3.5 m^3^/s) when we could wade into the rivers. At each site, we stretched a string horizontally from the metal peg (water level at bankfull flows) and measured the distance from the present water surface (the difference in water level) with a meter stick. Subsequently, we measured water depth at intervals of 1–3 m along one transect across the river with a meter stick and measured river width and water surface slope with a laser distance meter.

Finally, we calculated the channel cross-sectional area and wetted perimeter based on water depth at bankfull flow levels, which was obtained by adding the difference in water level to the water depth at low flows.

Average shear stress at bankfull flow level for the cross-section was calculated as 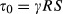
(2)where *γ* is the specific weight of water (1 g/cm^3^ at 20°C), *R* is the hydraulic radius (channel cross-sectional area/wetted perimeter), and *S* is the water surface slope (Lorang and Hauer 2003).

To estimate the range of particle sizes displaced during bankfull flows, we calculated critical particle size (*D*_crit_). Critical particle size is the largest particle diameter dislodged under a given shear stress, and it can theoretically be estimated by equating shear stress with critical shear stress (Death and Winterbourn 1994). Critical shear stress (τ_crit_) was defined in our work according to the Shields diagram (Lorang and Hauer 2003): 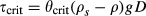
(3)

Equating τ_0_ with equation 2 and solving for *D* yields 
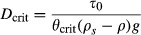
(4)where *θ*_crit_ is the Shields entrainment function (Lorang and Hauer 2003), *ρ*_*s*_ is the density of the particle being entrained, *ρ* is the density of water, *g* is the force of gravity, and *D* is the particle diameter. As the Shubuto River system is composed of high-gradient streams (*S *>* *0.002), the value of 0.02 was used for *θ*_crit_ as advised by Lorang and Hauer (2003). For other parameters, we used the following values: *g *=* *980 cm/s^2^, *ρ*_*s*_ = 2.65 g/cm^3^, and *ρ *= 1.0 g/cm^3^.

Equation 2 represents “average” hydraulic force to wash out bed materials at each sampling site, but mussels might exploit local refugia within a sampling site (Steuer et al. 2008; Daraio et al. 2010a). To better address this possibility, we estimated local variation in shear stress within a sampling site based on the local water depth (Lorang and Hauer 2003; Asami et al. 2012): 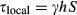
(5)where *h* is the local water depth at bankfull flow level at each measuring point for the cross-section. We refer to maximum values of τ_local_ at each sampling site as τ_max_, while referring to minimum values as τ_min_. Corresponding values of *D*_crit_ (either *D*_max_ or *D*_min_) was estimated by replacing τ_0_ with either τ_max_ or τ_min_ in equation 4.

### Habitat quality under low-flow conditions

Physical attributes commonly linked to local abundance of freshwater mussels (water depth, current velocity, and bottom substrate) were measured in each quadrat concurrent with the mussel sampling. We measured water depth with a meter stick and current velocity with a flow meter (VE20, VET-200-10PII; KENNEK, Tokyo, Japan). We visually estimated the coverage of three types of substrate: sand (particle size < 2.0 mm), gravel (2.0–64 mm), and cobble (>64 mm). Percent sand cover was used in subsequent analyses because it was found to be particularly important for *M. laevis* (Terui et al. 2011). All measurements were taken before collecting mussels.

Three factors related to water quality (pH, turbidity, and dissolved O_2_) were measured at three points, including mid-channel and adjacent to each bank. We measured pH with a pH meter (D-55; Horiba, Kyoto, Japan), turbidity with a turbidity meter (TR-30; Kasahara Chemical Instruments, Kuki City, Japan), and dissolved O_2_ with a dissolved O_2_ meter (model 550A; YSI, Kawasaki City, Japan).

Hyporheic exchange, which involves subsurface flow through the streambed, might be an important determinant of mussel distribution because juvenile recruitment is linked to streambed sedimentation (e.g., Geist and Auerswald 2007). Unfortunately, obtaining direct measures of hyporheic exchange across our study system was impractical. Therefore, we used a sinuosity index as a proxy variable, reasoning that greater hyporheic exchange would occur in association with the lateral irregularities of sinuous channels (Kasahara and Wondzell 2003; Isaak et al. 2007). The sinuosity index was calculated as the stream length through a sampling site (100 m) divided by the straight-line distance between endpoints (Fukushima 2001). Higher values in this index represent higher lateral irregularities.

After averaging duplicated measurements for each site, we performed principal components analysis (PCA) to reduce the dimensionality of these attributes, as local habitat variables were correlated with each other. The cumulative contribution of the first two principal components (PCs) was 0.62 and changed little when including additional PCs; therefore, we used only the first two PCs (“habitat quality” hereafter) for subsequent analyses (Table 1). Turbidity and dissolved O_2_ were not included in the PCA because their variation among sampling sites was negligible (turbidity < 1.0 NTU; dissolved O_2_ >95% in degrees of saturation).

**Table 1:** Axis loadings from principal components analysis used to summarize habitat quality attributes.

Covariate	PC1	PC2
Water depth	−0.59	0.06
Current velocity	0.51	0.09
% sand	−0.50	−0.47
pH	−0.34	0.53
Sinuosity index	0.10	−0.70
Variance explained (%)	38.8	23.7

### Statistical analyses

All analyses were conducted with R version 2.15.3 (R Development Core Team 2013). All datasets were analyzed with a generalized linear mixed model (GLMM), in which the response variable was total mussel abundance per site with a negative binomial error distribution and log-link function (package: glmmADMB). We incorporated individual river sections bounded by neighboring confluences as a random effect to account for differences among river sections that were not captured by our habitat quality measurements (Bolker et al. 2009). We also included sampling year as a random effect to account for random variation among sampling years.

Explanatory variables were the UDR, NUC (1, 2, or 3 km), proximity to downstream reproductive subpopulations, shear stress (average values derived from Equation 2; see above), and habitat quality (PC1 and PC2; see above). Average shear stress was used in this analysis because it was strongly correlated with values of maximum and minimum shear stress (Pearson's *r* > 0.95 for both). We also included Strahler stream order (Strahler 1957) as a control variable in the models to account for any effect of stream size, which is known to influence mussel species richness and composition (e.g., Strayer 1983). All continuous variables were standardized by subtracting mean values and by dividing by two standard deviations, allowing us to compare the effect size among explanatory variables.

Stream order was positively correlated with the UDR (Pearson's *r* = 0.7). Therefore, we used residuals of a linear relationship fitted between stream order and the UDR to avoid multicollinearity. The residuals provide a relative measure of the stream order, independent of the UDR, in which positive values reflect a greater stream order than expected for a given UDR, whereas negative values reflect a lower stream order than expected. After this procedure, variance inflation factors for all explanatory variables showed values of <4.0, indicating little influence of multicollinearity (Miles and Shelvin 2001).

We used Akaike's information criterion (AIC; Burnham and Anderson 2002) to select the best-fit regression model. We compared performance among models with different NUC distance classes (1, 2, or 3 km) and considered that with the lowest AIC to be the best model. In general, models with an AIC ≤2.0 different from the best model have substantial support for explaining the data, whereas models with a difference in AIC of >4.0 have considerably less support (Burnham and Anderson 2002).

## Results

### Spatial distribution of reproductive subpopulations

In total, we surveyed 88.7 km of riverine network in 34 streams. We found reproductive subpopulations in the Shubuto mainstem, Neppu, and Raiba rivers, and their upstream range limits were located 29 km, 21 km, and 30.5 km, respectively, from the mouth of the mainstem (Fig. 2). Reaches (0.5 km) with reproductive subpopulations were continuously distributed in the lower mainstem, but became sparser with distance upstream (Fig. 2). Reproductive subpopulations were evenly distributed in the Neppu River, but occurred only a short distance into the Raiba River (Fig. 2). Each reproductive subpopulation had a longitudinal extent of <20 m and was patchily distributed within the reach.

### Environmental conditions among quantitative sampling sites

Environmental conditions at low flows among the 57 sampling sites (Fig. 2) varied as follows (Table 2): river width (2.0–34.5 m), water depth (0.06–0.94 m), current velocity (0.04–0.68 m/s), and pH (7.0–8.1). Gravel was the dominant substrate type followed by cobble and sand (Table 2). The sinuosity index varied from 1.0 to 1.99 (Table 2). The river system was composed of high-gradient streams, with water surface slope ranging from 0.002 to 0.022 m/m. Average shear stress at bankfull flow (τ_0_) varied from 0.05 to 1.72 g/cm^2^, while critical particle size ranged from 1.4 to 53.3 mm (Table 2), indicating that a wide range of particle sizes can be displaced in less stable reaches under bankfull flow conditions. However, there was significant variation in local shear stress within a sampling site (Table 2). Maximum shear stress (τ_max_) widely varied from 0.06 to 2.44 g/cm^2^, whereas minimum shear stress (τ_min_) had a narrower range (0.03–1.21 g/cm^2^). Maximum and minimum values of critical particle size at each sampling site varied in concordance with the local variation in shear stress (1.9–75.4 mm for *D*_max_ and 0.8–37.4 mm for *D*_min_).

**Table 2:** Environmental conditions among quantitative sampling sites.

Variable	Mean	SD	Range
Wetted width (m)	10.8	8.2	2.0–34.5
Water depth (m)	0.34	0.17	0.06–0.94
Current velocity (m/s)	0.30	0.14	0.04–0.68
pH	7.6	0.3	7.0–8.1
% sand	13.5	11.2	0–56.7
% gravel	44.6	17.8	13.3–88.8
% cobble	41.4	22.7	0–85.8
Sinuosity index	1.04	0.13	1.0–1.99
Water surface slope (m/m)	0.008	0.006	0.002–0.023
τ_0_(g/cm^2^)	0.38	0.32	0.05–1.72
τ_min_ (g/cm^2^)	0.24	0.22	0.03–1.21
τ_max_ (g/cm^2^)	0.55	0.48	0.06–2.44
*D*_crit_ (mm)	11.7	10.0	1.4–53.3
*D*_min_ (mm)	7.7	6.8	0.8–37.4
*D*_max_ (mm)	17.1	14.7	1.9–75.4

### Settled subpopulation size and shell length distribution

Of 57 sampling sites, we found 26 settled subpopulations including small, nonreproductive ones detected in the Nakano and Horonai rivers (Fig. 2). Settled subpopulations varied greatly in size: the means of total, adult (≥50 mm in shell length), and juvenile (<50 mm) abundance were 100.4 ± 172.3 (mean ± SD), 50.0 ± 80.8, and 54.4 ± 95.2, respectively. Adult and juvenile abundance at each site were strongly correlated (Pearson's *r *= 0.92, *P* < 0.01), suggesting that local recruitment occurred. No subpopulations were found outside of the distribution range of potential immigrants (Fig. 2).

**Figure 3:**
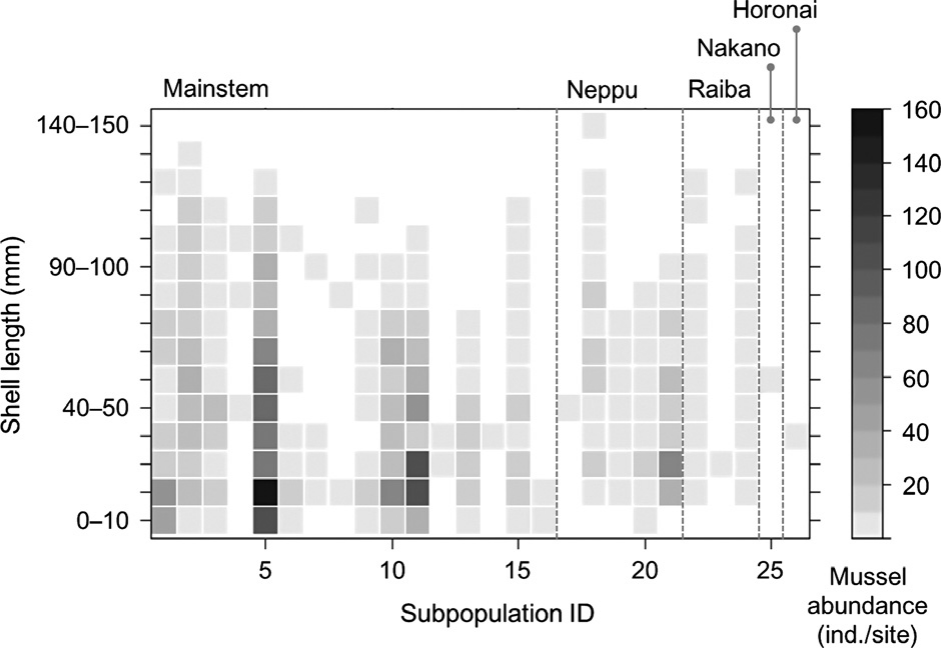
Shell length distribution of 26 settled subpopulations. The *x*-axis represents the subpopulation ID, corresponding to the numbers shown in Fig. 1 and is ordered by the distance from each of the stream mouths. Each cell indicates mussel abundance within a given size class for each subpopulation.

The distribution of shell length differed substantially among settled subpopulations. Large subpopulations included a wide range of size classes with many juveniles (Fig. 3). In contrast, small settled subpopulations were composed exclusively of a few juveniles (subpopulation ID = 12, 14, 17, 23, and 26), and such subpopulations were often found in branch tips of the distribution range of potential immigrants (subpopulation ID = 16, 23, and 26; see Figs. 2 and 3). Juvenile mussels occurred in a wider range of habitats than adult mussels (Fig. 3), implying that water- and host-mediated dispersals are functioning in this system.

### Factors affecting the settled subpopulation size

**Figure 4:**
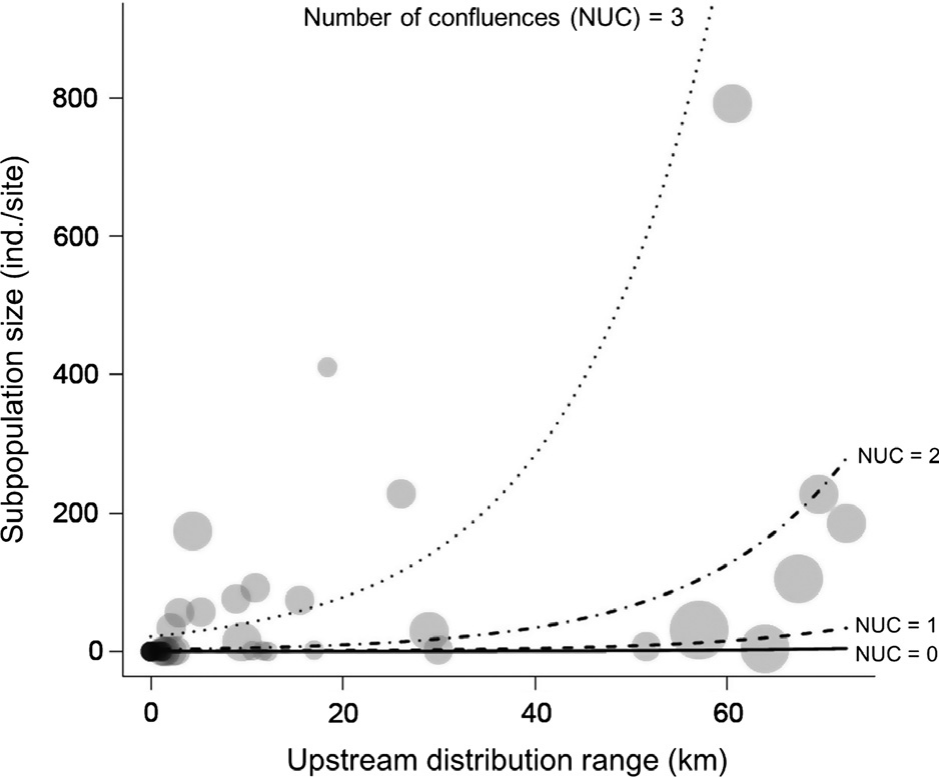
Strong positive effects of the upstream distribution range (UDR) of potential immigrants and the number of upstream confluences (NUC) on *Margaritifera laevis* subpopulation size. Solid, broken, broken-dotted, and dotted lines (NUC = 0, 1, 2, and 3, respectively) indicate predicted values from the best model (GLMM with negative binomial error distribution) explaining the subpopulation size. Gray bubbles indicate observed values of subpopulation size, and the size of bubbles is proportional to the NUC. The values of all predictors except the UDR and NUC are fixed at their median values to isolate the partial contribution of these variables to the response variable.

**Figure 5:**
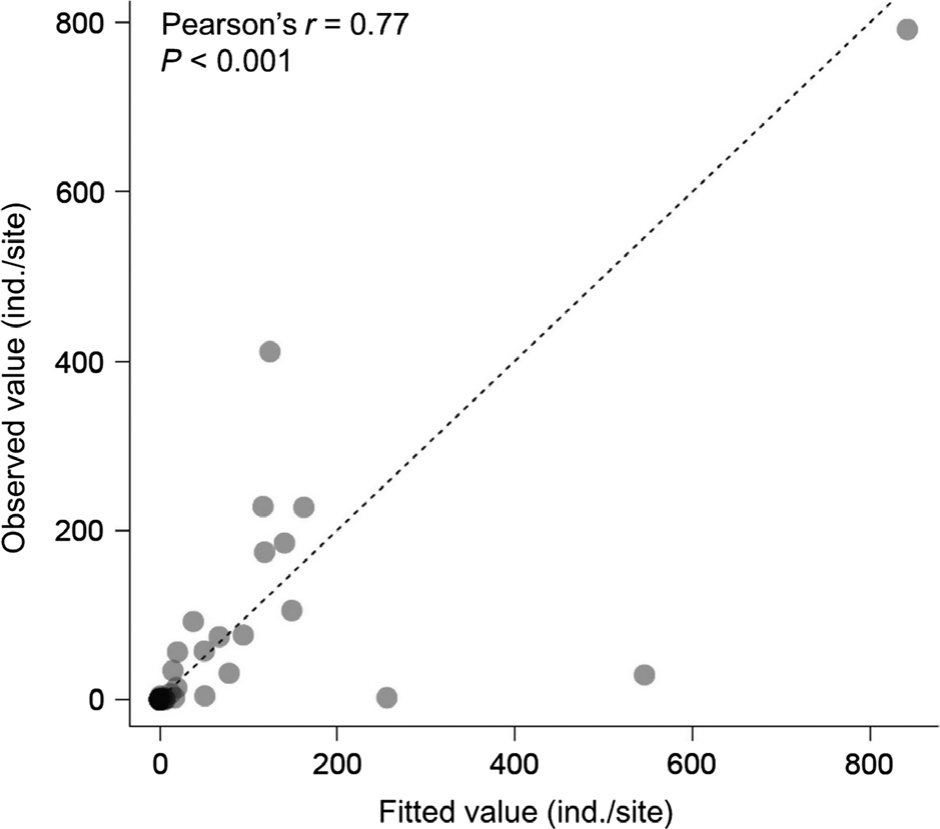
The best model well explained *Margaritifera laevis* subpopulation size as evidenced by the relationship between predicted and observed values of subpopulation size (ind./site). The broken line indicates a 1:1 relationship.

When total *M. laevis* abundance (a proxy for the settled subpopulation size) was used as a response variable, the model including the NUC at a scale of 2 km had the lowest AIC (AIC = 327.3 at 1 km, 319.8 at 2 km, and 324.8 at 3 km), indicating that model performance was highest at this scale. This model revealed that the UDR and NUC (proxies for upstream immigrant sources) strongly predicted total *M. laevis* abundance: total abundance sharply increases with increasing UDR and NUC (Fig. 4). In contrast, little influence of proximity to downstream reproductive subpopulations (a proxy for downstream immigrant sources; Table 3) was observed. Shear stress at bankfull flows had a negative effect on total *M. laevis* abundance, whereas habitat quality at low flows had little influence (Table 3). Stream size had a strong positive effect and its 95% confidence interval did not include zero (Table 3). The best model well explained observed values of total abundance (Fig. 5) as evidenced by the Pearson's correlation coefficient between predicted and observed values (Pearson's *r* = 0.77, *P* < 0.001).

**Table 3:** Parameter estimates for the best model predicting the total abundance (ind./site).

Explanatory variable	Coefficient	SE	95% CI
**UDR**	**2.69**	1.29	**0.16–5.21**
**NUC_2 km**	**3.96**	1.36	**1.29–6.62**
Proximity	−0.37	1.09	−2.51–1.78
**Shear stress**	**−2.33**	0.93	**−4.16–−0.50**
Habitat quality_PC1	0.69	0.62	−1.82–3.21
Habitat quality_PC2	2.31	0.85	−0.99–5.60
**Stream order**^1^	**4.73**	1.49	**1.82–7.64**

Coefficient, standardized partial regression coefficient; SE, standard error; 95% CI, 95% confidence interval; UDR, upstream distribution range of potential immigrants; NUC, the number of upstream tributaries; Proximity, proximity to downstream reproductive subpopulations.

Bold faces indicate variables whose 95% CI did not include zero.

1 Residuals of the fit linear relationship between Strahler stream order and UDR.

## Discussion

Our study revealed that the settled subpopulation size of *M. laevis* is greatly influenced by UDR and NUC, which are proxy variables for the number of potential immigrants from upstream. This influence was consistent even after accounting for the effects of downstream immigrant sources (proximity to downstream reproductive subpopulations) and other potential covariates (substrate stability, local habitat quality, and stream size). These results support our hypothesized metapopulation dynamics, in which unsettled mussels moving down the river act as major sources of immigrants for settled subpopulations downstream.

Most metapopulation studies have assumed directionally symmetric dispersal, an assumption frequently applied to salmonids whose swimming ability is sufficient to overcome river currents (Dunham and Rieman 1999; Koizumi and Maekawa 2004; Isaak et al. 2007). In symmetric systems, larger subpopulations are well recognized to be the likely dominant sources of immigrants to other subpopulations (Hanski 1999) and often take priority in conservation efforts (e.g., MacPherson and Bright 2011). However, this general notion can be violated for species under the strong influence of asymmetric dispersal. Our finding suggests that under asymmetric dispersal, certain portions of a population's overall range—in this case upstream subpopulations of the riverine mussel *M. laevis*—may be disproportionately important as immigrant sources, irrespective of actual subpopulation size. Indeed, subpopulation size decreased with increasing upstream distance, despite these subpopulations functioning as primary immigrant sources. To our knowledge, this study provides the first empirical evidence that asymmetric dispersal can structure a riverine metapopulation of a less-mobile organism.

Hydraulic conditions suggest that the positive effect of UDR is likely a reflection of increased emigration from settled source subpopulations upstream. Minimum shear stress at bankfull flows was high enough to dislodge particles up to ∼37.4 mm in diameter, which corresponds to the size of *M. laevis* up to ∼10 years of age (Akiyama and Iwakuma 2009). Given the frequency of bankfull flows in this system (2–5 times per year; Terui 2014), mussels probably are occasionally subjected to downstream drift for at least the first several years of their lives. In fact, after a flood event (e.g., bankfull flows), mussels colonized a newly created artificial stream whose inlet directly connects with the mainstem (Terui 2014). Therefore, settled subpopulations with a longer UDR may benefit from an increased cumulative supply of immigrants from upstream.

The positive effect of the NUC is understandable given the dispersal pattern of host fish. *Oncorhynchus masou masou* infected with glochidia preferentially move to cooler tributaries during summer in the Shubuto River system (Terui et al. 2014), which may cause the aggregation of tiny juvenile *M. laevis* in tributaries rather than the mainstem. Juvenile mussels can drift great distances (∼7 km; see Morales et al. 2006; Daraio et al. 2010b) before settling even in low-gradient streams, and in the relatively high-gradient Shubuto River system (slope > 0.002), juvenile *M. laevis* would likely drift significantly downstream before settling. Thus, the small settled subpopulations found in branch tips of the distribution range of potential immigrants may be but shadows of a dispersal process mediated by host fish and river currents.

Shear stress under bankfull flow conditions, but not habitat quality under low-flow conditions, had a strong effect on the settled *M. laevis* subpopulation size. This result is consistent with previous studies showing that streambed stability under high-flow conditions limits mussel abundance and species richness at various spatial scales (Morales et al. 2006; Allen and Vaughn 2010; Daraio et al. 2010a). Low shear stress during sudden water surges may not only reduce emigration of settled mussels from mussel beds but also promote the colonization of unsettled mussels. Therefore, in physically stable areas under flood conditions, mussels can settle for long enough to grow significantly.

Although we attempted to address this issue by including a wide array of habitat attributes under different flow conditions, it is possible that the observed spatial patterns may have been governed by unmeasured factors that were only implicitly included in the statistical models as random effects. This remains largely unavoidable, as the habitat requirements of unionoid mussels are still poorly understood (see Strayer 2008).

This study highlights the importance of passive, water-mediated dispersal, but host-mediated dispersal also plays a role in *M. laevis* metapopulation dynamics. Mussels are primarily sessile or passive dispersers, so upstream range expansion is virtually impossible without host-mediated dispersal (Terui et al. 2014). This process is critical to the long-term persistence of mussel metapopulations—once a new upstream “frontier” is established by immigration from the previous upstream frontier, the new subpopulation may play a pivotal role in supporting downstream subpopulations in the short term. This argument is broadly applicable given that many lotic organisms, particularly species with limited motility, share life histories analogous to that of *M. laevis*. For example, aquatic insects are readily transported by river currents (Williams and Williams 1993) following upstream dispersal in their winged adult life stage (Macneale et al. 2005; reviewed by Smith et al. 2009). *Margaritifera laevis* is probably an extreme case of asymmetric dispersal because it is both sessile and long-lived, and its population structure and dynamics may vary depending on the magnitude of asymmetry (Vuilleumier et al. 2010). Further empirical studies focusing on this issue are needed, and such information would provide greater insight into riverine metapopulation dynamics.
